# Ethnicity and skin autofluorescence-based risk-engines for cardiovascular disease and diabetes mellitus

**DOI:** 10.1371/journal.pone.0185175

**Published:** 2017-09-20

**Authors:** Muhammad Saeed Ahmad, Torben Kimhofer, Sultan Ahmad, Mohammed Nabil AlAma, Hala Hisham Mosli, Salwa Ibrahim Hindawi, Dennis O. Mook-Kanamori, Katarína Šebeková, Zoheir Abdullah Damanhouri, Elaine Holmes

**Affiliations:** 1 Drug Metabolism Unit, King Fahad Medical Research Center, King Abdulaziz University, Jeddah, Saudi Arabia; 2 Biomolecular Medicine, Department of Surgery and Cancer, Faculty of Medicine, Imperial College London, South Kensington, London, United Kingdom; 3 Cardiology Unit, Department of Medicine, King Abdulaziz University Hospital, Jeddah, Saudi Arabia; 4 Department of Medicine, Faculty of Medicine, King Abdulaziz University, Jeddah, Saudi Arabia; 5 Department of Haematology, Faculty of Medicine, King Abdulaziz University, Jeddah, Saudi Arabia; 6 Department of Primary Care/Public Health and Clinical Epidemiology, Leiden University Medical Center, Leiden, The Netherlands; 7 Institute of Molecular Biomedicine, Faculty of Medicine, Comenius University, Bratislava, Slovakia; 8 Department of Pharmacology, Faculty of Medicine, King Abdulaziz University, Jeddah, Saudi Arabia; Medical University of Gdańsk, POLAND

## Abstract

Skin auto fluorescence (SAF) is used as a proxy for the accumulation of advanced glycation end products (AGEs) and has been proposed to stratify patients into cardiovascular disease (CVD) and diabetes mellitus (DM) risk groups. This study evaluates the effects of seven different ethnicities (Arab, Central-East African, Eastern Mediterranean, European, North African, South Asian and Southeast Asian) and gender on SAF as well as validating SAF assessment as a risk estimation tool for CVD and DM in an Arabian cohort. SAF data from self-reported healthy 2,780 individuals, collated from three independent studies, has been linear modelled using age and gender as a covariate. A cross-study harmonized effect size (Cohens’*d*) is provided for each ethnicity. Furthermore, new data has been collected from a clinically well-defined patient group of 235 individuals, to evaluate SAF as a clinical tool for DM and CVD-risk estimation in an Arab cohort. In an Arab population, SAF-based CVD and/or DM risk-estimation can be improved by referencing to ethnicity and gender-specific SAF values. Highest SAF values were observed for the North African population, followed by East Mediterranean, Arab, South Asian and European populations. The South Asian population had a slightly steeper slope in SAF values with age compared to other ethnic groups. All ethnic groups except Europeans showed a significant gender effect. When compared with a European group, effect size was highest for Eastern Mediterranean group and lowest for South Asian group. The Central-East African and Southeast Asian ethnicity matched closest to the Arab and Eastern Mediterranean ethnicities, respectively. Ethnic and gender-specific data improves performance in SAF-based CVD and DM risk estimation. The provided harmonized effect size allows a direct comparison of SAF in different ethnicities. For the first time, gender differences in SAF are described for North African and East Mediterranean populations.

## Introduction

The enzymatic cross-linking of carbohydrates to other organic molecules, e.g., proteins, is commonly referred to as glycosylation and is one of the most important post-translational modifications, essential for human cell signaling and metabolism. Usually less commonly known is the non-enzymatic and therefore less specific reaction equivalent, which was first described in 1912 and named eponymously after its discoverer Louis-Camille Maillard. The Maillard reaction takes place in multiple steps and eventually leads to the irreversible formation of advanced glycation end products (AGEs). Generally, the formation of AGEs is a slow process which occurs physiologically *in vivo*, with accumulation of AGEs in tissues with slow renewal rates (e.g., retina, dermis). In situations of metabolic and oxidative stress, however, accelerated AGE accumulation rates have been described, such as in individuals with cardiovascular disease (CVD) [1,2] and diabetes mellitus (DM) [3,4]. The fairly quick (seconds) and non-invasive assessment of skin autofluorescence (SAF) has been demonstrated to serve as a surrogate marker of tissue accumulation of AGEs [5,6] and recent efforts have been undertaken to develop stratification tools for the clinical assessment of a patient's risk for cardiovascular disease (CVD) [7–9] and diabetic complications [10–12].

In previous studies, we have shown that SAF baseline intensity is ethnicity-specific with significant gender effects present in a Middle Eastern but not in European populations [13–15]. These findings suggest that the currently available SAF-based risk scoring models, which have been established with mainly Caucasian [16] and Asian populations [17,18], cannot be correctly applied to non-European ethnicities. Thus, a detailed characterization of ethnicity and gender driven differences in SAF is required to improve the models’ accuracies.

The purpose of the present study is two-fold: First, to provide a cross-study harmonized effect size (the mean standardized difference) for ethnicity and gender differences in SAF levels, which allows to directly compare gender and ethnicity effects in different cohorts. Second, to validate the utility of SAF assessment for the screening and monitoring of CVD and DM in an Arab population, monitoring beyond the previous characterization of SAF values in a 'healthy' Saudi Arabian population.

## Materials and methods

### Data

The present study combines 2,780 SAF measurements for self-reported healthy individuals from three independent data sets, [13–15] (S1 Table). Additional SAF measurements have been newly collected at King Abdulaziz University Hospital from 235 adults suffering either from CVD, diabetes mellitus (DM) or both, between September 2015 and September 2016. For CVD cohort, we recruited previously diagnosed CVD patients with one or more of the following pathologies: Ischemic heart disease, myocardial infarction, cardiac failure, angina and atherosclerosis (see shared dataset). Those patients had echocardiography and/or coronary angiography as a standard procedure, and the results were evaluated by an experienced interventional cardiologist. Diabetes patients were selected according to WHO diagnosis criteria with fasting venous plasma glucose level of equal or greater than 7 mmol/L and/or 2-h post glucose load of equal or greater than 11.1 mmol/L at initial diagnosis. Only patients with established medical history and on medication for the specific disease were recruited from the specialty outpatients’ clinics of King Abdulaziz University Hospital. Cancer patients, pregnant women and subjects with kidney diseases were excluded from the study as well as subjects with skin type V-VI on Fitzpatrick scale. The study was approved by the Research Ethics Committee of King Abdulaziz University and written informed consent was obtained from all participants.

SAF values were measured at the right forearm using the AGE Reader® (DiagnOptics Technologies B.V., Groningen, the Netherlands, Software V 2.3.0.7) as described in detail previously [13]. A questionnaire was used to survey demography, ethnicity, country of birth, life style and medical history information, and physical measurements such as weight, waist circumference, hip circumference, blood pressure were also recorded according to a standard protocol.

### Linear modelling and univariate statistics

Linear regression models were established for each geographic cluster, using SAF as the dependent variable and age [years] and gender as independent variables:

$$SAF=b_{0}+b_{1}*age+b_{2}*gender$$

With *b*_*1*_ and *b*_*2*_ representing the coefficients for age and gender, respectively, whereas the gender variable was binary coded with 1 (male) or 0 (female). Ethnicity was determined by the participants’ country of birth, rather than current residence. To compare the different ethnicities directly, an additional linear model was established that includes the variable ethnicity and an interaction term of gender and ethnicity:

$$SAF=b_{0}+b_{1}*age+b_{2}*gender+b_{3}*ethnicity+b_{4}*gender:ethnicity$$

From this model, p values were extracted for each estimated model coefficient. Model residuals were assessed graphically by plotting the residuals quantiles against the quantiles of a theoretical normal distribution with identical mean and standard deviation (this plot type is commonly referred to as Q-Q plot). Model residuals showed approximate normal distribution with no obvious patterns or trends, indicating that linear regression modelling is an appropriate choice for modelling SAF in the context of age, ethnicity and gender.

Normally and non-normally distributed variables were expressed as mean (+/- standard deviation) and median (range), respectively. Statistical group comparison was performed with the t-test for normally distributed variables and the Mann-Whitney U test, as a non-parametric alternative, for non-normally distributed variables. All presented p values represent two-tailed tests. Group comparisons were deemed as statistically significant if the resulting p value was below a threshold of 0.05.

### Effect size

To assess the magnitude and strength of a relationship between two variables, e.g., SAF in men and women, the standardized mean difference (MSD), also known as Cohen’s *d*, was chosen as an effect size measure. The Cohen’s *d* is defined as the difference between two group means ($\bar{x}$), normalized by the pooled standard deviation (*s*):

$$d=\frac{{\bar{x}}_{a}-{\bar{x}}_{b}}{s_{ab}}$$

Whereas *s* denotes the pooled standard deviation of group *a* and *b*, defined as

$$s_{ab}=\sqrt{\frac{s_{a}^{2}+s_{b}^{2}}{2}}$$

With *s*^*2*^ referring to the sample variances. In general, Cohen’s *d* values below an absolute value of 0.2 are considered as negligible, whereas absolute values below 0.5 and 0.8 are considered as small and intermediate effect sizes, respectively. An absolute Cohen’s *d* value of 0.8 or higher is considered as a large effect size.

### Performance assessment of CVD risk engine

SAF-based CVD risk engines were applied to individuals with CVD, DM or mixed pathologies, resulting in the stratification of patients into three risk groups (no risk, elevated risk, high risk) as described previously elsewhere [13]. Accuracy has been chosen as a measure of performance, defined as

$$Accuracy=\frac{TP+TN}{n}$$

with *TP* representing the number of individuals suffering from CVD, DM or both and stratified into risk group I or higher; TN indicates the number of healthy individuals stratified into risk group 0 and *n* represents the total number of participants.

All computation was performed with the statistical programming language R (V 3.2.2) [19] running on a Mac OSX machine.

## Results

### Baseline characteristics

**Fig 1:**
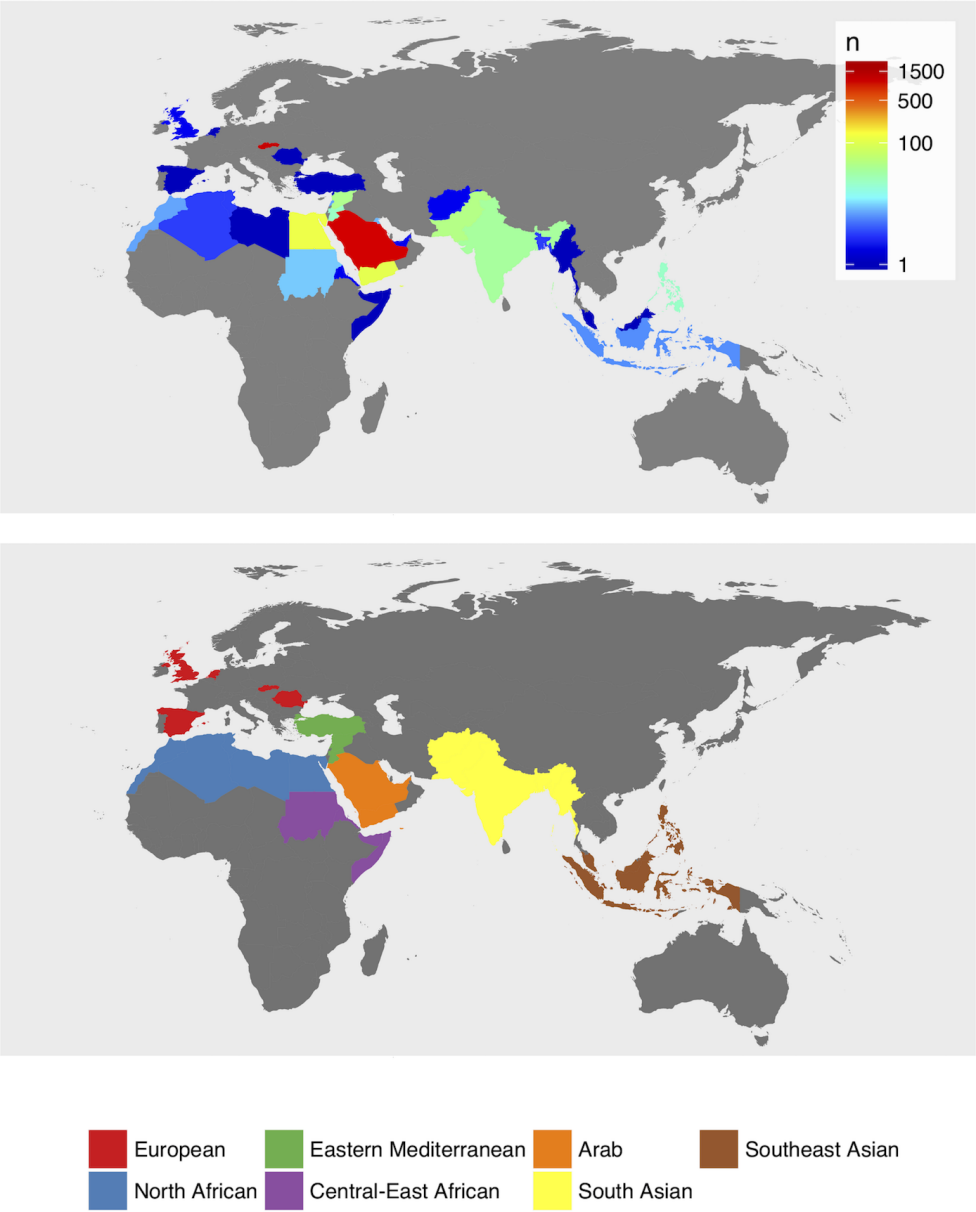
Ethnicities and sample numbers for all study participants (upper panel); geographic stratification for subsequent statistical analysis (lower panel).

The present study includes SAF measurement performed in Saudi Arabia, Qatar and Slovakia for a total of 3,015 individuals representing 36 different countries (Fig 1, S1 Table). A subset of 2,780 individuals self-reported the absence of disease (further referred to as healthy), with a median age of 35 years (18–81 years), and a median SAF and Skin reflectance (SR) value of 1.70 AU (0.75–4.84 AU) and 0.12 (0.04–0.50), respectively (S2 Table). The remaining 235 individuals suffered from at least one pathology that is associated with increased SAF intensities, that is CVD (n = 50), DM (n = 111) or mixed phenotypes (DM+CVD, n = 74). The median age in this group was 46 years (18–90 years), with a median SAF value of 2.80 AU (1.20–6.92 AU) and a median SR value of 0.08 (0.06–0.22) (S2 Table). The gender distributions of both cohorts were approximately the same with a respective female:male ratio of 1.1 and 0.9 for the healthy and disease cohort, respectively.

### Characterization of SAF in different ethnicities and gender

For further analysis, individuals were stratified into the following geographic clusters based on ethnicity or, if that was unavailable, country of birth: European (n = 1,177), North African (n = 137), Eastern Mediterranean (n = 92), Central-East African (n = 12), Arab (n = 1,181), South Asian (n = 152) and Southeast Asian (n = 29) (lower panel of Fig 1, S3 Table).

Linear modelling of SAF vs age of healthy individuals, including gender as a covariate, showed that over the entire age range, the North African population, comprising mainly Egyptians (90%), had the highest SAF values (b_0_ coefficient = 1.55, p value = 2.1 x 10^−14^) with a pronounced gender effect, men displayed on average lower SAF values than women (male gender coefficient = -0.61, p value = 1.8 x 10^−12^) (Table 1). Comparable but slightly lower SAF profiles and gender effects have been observed for Arab and Eastern Mediterranean individuals, with b_0_ coefficients of 1.40 (p value < 2 x 10^−16^) and 1.44 (p value = 1.4 x 10^−11^), respectively and a male gender coefficient of -0.41 for both ethnicities (p value < 2.0 x 10^−16^ and 1.0 x 10^−4^ for Arab and Eastern Mediterranean ethnicity, respectively). In contrast, the European cohort, comprising mainly Slovak individuals (99%), showed lowest SAF signals (b_0_ coefficient = 0.86, p value < 2 x 10^−16^) with a negligible small gender effect (male gender coefficient -0.07, p value = 3.4 x 10^−8^) (Table 1), as noted before [5], [14]. The South Asian cohort showed a SAF profile with a very similar gender effect to the Arab and Eastern Mediterranean population (male gender coefficient -0.41, p value = 9.4 x 10^−6^), but with SAF intensities between those of European and Eastern Mediterranean populations (b_0_ coefficient = 1.13, p value = 2.8 x 10^−10^) and slightly steeper increasing SAF values with age (age coefficient = 0.027, p value = 3.6 x 10^−12^). Linear model residual and leverage plots indicated no model irregularities (S1 Fig).

**Table 1:** SAF model comparison for different ethnic groups.

Ethnicity	Model coefficients			R^2^	Cohen’s *d* effect size^§^
	b_0_	Age	Male		
Arab (n = 1,181)	1.40*	0.022*	-0.41*	0.33	1.3
**East. Mediterranean (n = 92)**	1.44*	0.021*	-0.41*	**0.31**	**1.5**
**European (n = 1,177)**	0.86*	0.020*	-0.07*	**0.48**	**-**
**North African (n = 137)**	1.55*	0.021*	-0.61*	**0.39**	**1.4**
**South Asian (n = 152)**	1.13*	0.027*	-0.41*	**0.31**	**1.2**

^§^ compared to European population after adjusting for age and gender

* p value < 0.01.

When comparing age and gender-adjusted SAF profiles across ethnicities, using the European population as a reference, the highest effect size was observed for Eastern Mediterranean populations (Cohen’s = 1.5), closely followed by North African (Cohen’s d = 1.4), Arabian Peninsula (Cohen’s d = 1.3) and South Asian (Cohen’s d = 1.2) populations (Table 1). The same trends have been observed for a linear model obtained by including the variable ethnicity and an ethnicity:gender interaction term (see S1 Text).

Since the number of individuals with Central-East African and Southeast Asian ethnicity was low (n<50), we abstained from training separate linear models for these cohorts; however, predicting SAF intensities of individuals with Central-East African and South Asian ethnicities with age and gender using the established linear models, indicated that the Arab and Eastern Mediterranean ethnicities provided the best fit with the smallest residual variation, with R^2^ values of 0.60 and 0.56, respectively.

### Effect of DM and CVD on SAF in an Arab cohort

**Fig 2:**
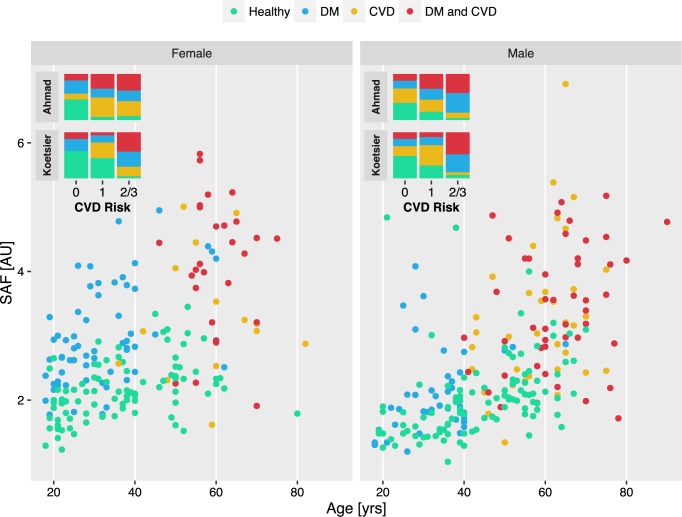
Comparison of SAF intensities between healthy individuals and CVD patients with and without diabetes in an Arab cohort. Insets show the distribution of risk groups as calculated with the established risk engine that is implemented in the AGE-Reader apparatus and the adjusted risk scheme for Middle Eastern populations, both described in Ahmad *et al*. [13].

Comparing SAF intensities between individuals suffering from DM, CVD or mixed pathologies to those from an age, gender and BMI matched healthy individuals in an Arab cohort, showed that SAF values were significantly increased in all tested disease groups (Table 2), with an effect size ranking of DM + CVD, CVD and DM (Cohen’s *d* = 1.4, 1.1 and 1.0, respectively). Interestingly, the SAF gender difference was also observed in disease phenotypes with higher effect sizes in women in the DM disease group (Cohen’s d 1.6 vs 0.4 for women and men, respectively), as well as for the DM + CVD group (Cohen’s d 1.9 vs 1.2 for women and men, respectively). These differences were also visible when plotting SAF values against age (Fig 2).

**Table 2:** Associations of SAF with different disease phenotypes in an Arab cohort.

Disease Phenotype	n	p value*	Cohen’s *d* effect size*	Accuracy % (risk > 0)	
				Ahmad	Koetsier
**Diabetes (DM)**	**111**	**5.3 x 10**^**−15**^	**1.0**	**69**	**55**
Women	64	5.1 x 10^−14^	1.6	78	52
Men	47	2.0 x 10^−3^	0.4	57	60
**Cardiovascular Disease (CVD)**	**50**	**6.1 x 10**^**−7**^	**1.1**	**70**	**63**
** Women**	**15**	**5.7 x 10**^**−3**^	**1.1**	**66**	**63**
** Men**	**35**	**1.5 x 10**^**−5**^	**1.1**	**71**	**63**
**DM and CVD**	**74**	**5.5 x 10**^**−13**^	**1.4**	**78**	**66**
** Women**	**27**	**1.3 x 10**^**−7**^	**1.9**	**81**	**63**
** Men**	**47**	**4.5 x 10**^**−8**^	**1.2**	**76**	**68**

* derived from age and gender-adjusted SAF values.

Further, we applied the CVD risk engine for healthy Saudi populations [13] to the disease cohorts and compared its performance to the traditional CVD risk engine provided by AGE reader software version 2.3.07 that has been derived from a healthy Dutch cohort [16]. Overall, the accuracy of both risk schemes resembled the effect size ranking. The DM + CVD group reached an accuracy of 78% (81% and 76% for women and men, respectively) when using the risk engine for Saudi populations, compared to 66% (63% and 68% for women and men, respectively) when using the conventional risk engine (Table 2). The accuracy for the CVD group dropped to 70% for the Saudi risk engine, with higher accuracy in men than in women (71 vs 66, respectively) and to 63% with the standard AGE-Reader provided risk engine. The DM group reached an accuracy of 69% when predicted with the Saudi risk engine, and 55% with the conventional Dutch risk engine, with generally higher accuracy in women than in men. Conversely, using the Dutch risk engine a slightly higher accuracy was observed in men (Table 2).

## Discussion

The present study shows that CVD and DM risk assessment can be improved by using gender and ethnicity-specific SAF reference values in a Saudi population. Linear modelling of SAF over age for five different ethnicities showed that Arab and Eastern Mediterranean ethnicities were comparable, so that the earlier proposed CVD risk stratification scheme for a Middle Eastern cohort can be readily applied to Eastern Mediterranean individuals. The North African population showed slightly higher but comparable baseline SAF values than the Arab and Eastern Mediterranean ethnicities, however, the gender effect (higher SAF in females) was significantly more pronounced. The South Asian ethnicity was characterized by a similar gender effect as in Arabs and Eastern Mediterraneans but with lower baseline SAF values and a slightly steeper increase in SAF over time. In contrast, European ethnicities have lower SAF baseline values and a negligible gender effect. These results illustrate that the studied ethnicities show marked differences to the European population (range Cohen’s d = 1.2–1.5), indicating that the consideration of both, ethnicity and gender, in risk stratification schemes is likely to improve risk estimation in other ethnicities, as well as in other conditions that are linked to increased SAF values, such as coronary artery calcification [20], peripheral artery disease [8], cardiovascular prognosis [9,21], cardiovascular mortality [22], diabetic foot ulceration [23] and carotid artery intima media thickness [24].

Comparing the fit of the statistical models by means of coefficient of determination (R^2^, a statistical measure of how close the data are to the fitted regression line), shows that the European model has the best fit (R^2^ = 0.48). This may be due to the fact that our European model was built on a homogeneous cohort with mainly Slovak individuals. The model trained for Arab, North African, South Asian and Eastern Mediterranean ethnicities showed reasonable fits (range R^2^ = 0.31–0.39); however, a substantial degree of variation remains unexplained and requires further evaluation.

A major environmental factor that is likely to have profound effects on AGE accumulation is diet; however, only sparse data are available on this subject. Klenovics *et al*. reported that breastfed infants (consuming AGEs-poor diet) had lower SAF intensities than formula-fed infants (consuming AGEs-rich diet) [14]. Other dietary components that have been linked to SAF include caffeine, which has been positively associated with SAF, accounting for 4% of SAF variation [25], [26], and meat consumption, where lower SAF values have been observed for vegetarians in haemodialysis patients [27].

Besides environmental components, genetic factors are likely to contribute to the observed SAF phenotypes. While early studies with twin and sibling pairs have implicated that the genetic makeup is at least partly responsible for lens and skin fluorescence variations [28,29], a recent genome-wide association study identified a strong association of skin fluorescence with the N-acetyltransferase 2 (NAT2) acetylator genotype [30]. Another study has linked NAT2 with increased insulin resistance [31] that is prevalent in type 2 diabetes patients and is a known risk factor for CVD. It is thus possible that there exist interaction effects between environmental and genetic components that contribute to the individual SAF phenotype. The fact that the evolutionary diversity of NAT2 has been linked to dietary habits and ecoregions in sub-Saharan African populations [32] seems to further support this hypothesis.

The observed gender differences in SAF values are unlikely to be attributable to genetic contributions alone, as there are no reports about sex-chromosome linked association of SAF. Earlier, we speculated that these gender differences might be related to vitamin D deficiency with women tending to be habitually less exposed to sunlight. A recent report supports this hypothesis where SAF values were found negatively associated with serum vitamin D concentration [33]. Factors other than vitamin D, such as skinfold thickness, degree of forearm hairiness (men usually have more forearm hair and hair can quench light) might also play a significant role.

The European cohort in this study is mainly composed of Slovak individuals, for which generally lower SAF values have been reported when compared with the Dutch reference values [14]. This observation illustrates that that there might be differences in SAF intensity within the defined geographic clusters. The extent of SAF variations due to geographic location or cultural tradition is largely unknown and deserves further investigation.

This study is limited by its cross-sectional design and we recruited CVD and DM patients solely based on their medical history and medication use. We did not collect data about duration of diabetes and/or CVD. We included self-reported healthy subjects for the main analysis and did not screen for diabetes, CVD or renal function, therefore, results should be interpreted with caution. We do not have data for potential covariates such as smoking, hypertension and physical activity across all ethnic groups. However, in our previous study based on self-reported data, we found no significant association of smoking, hypertension and physical activity with SAF after adjusting for known confounding factors such as age, BMI, diabetes and SR [13].

Overall, our results emphasize the importance of conducting further large-scale studies comprising of multi-ethnic populations residing in different regions of the world to unravel genetic and/or environmental contributions in ethnic and gender difference in SAF and its role in CVD risk assessment.

## Conclusions

We show that a SAF-based risk engine developed by using ethnic and gender-specific data improves performance in CVD and DM risk estimation. Furthermore, we provide harmonized effect sizes for SAF that allows a direct comparison of ethnicities. We report gender difference in SAF values for the first time in North African and East Mediterranean populations.

## Supporting information

S1 Fig Linear model diagnostic plot. Residual plots (**left**): all residuals are centred around zero and show a fairly random pattern. The red line is a smoothed approximation of the running mean across 20 observations. Leverage plots (**right**): Data points with high leverage are coloured towards red in the colour spectrum.
(TIF)

S1 Table Cohort overview. (DOCX)

S2 Table Overall cohort baseline characteristics. (DOCX)

S3 Table Healthy cohort overview segmented by country of birth. (DOCX)

S1 Text Inclusion of ethnicity as a predictor variable. (DOCX)

## Abbreviations

AGE: Advanced glycation end product
SAF: Skin autofluorescence
SR: Skin reflectance
DM: Diabetes mellitus
CVD: Cardiovascular disease
BMI: Body mass index
AU: Arbitrary units
