# Supplementary figures and images for: Ethnicity and skin autofluorescence-based risk-engines for cardiovascular disease and diabetes mellitus
Source: PLoS One. 2017 Sep 20;12(9):e0185175. doi: 10.1371/journal.pone.0185175 (PMC5607192; doi:10.1371/journal.pone.0185175)

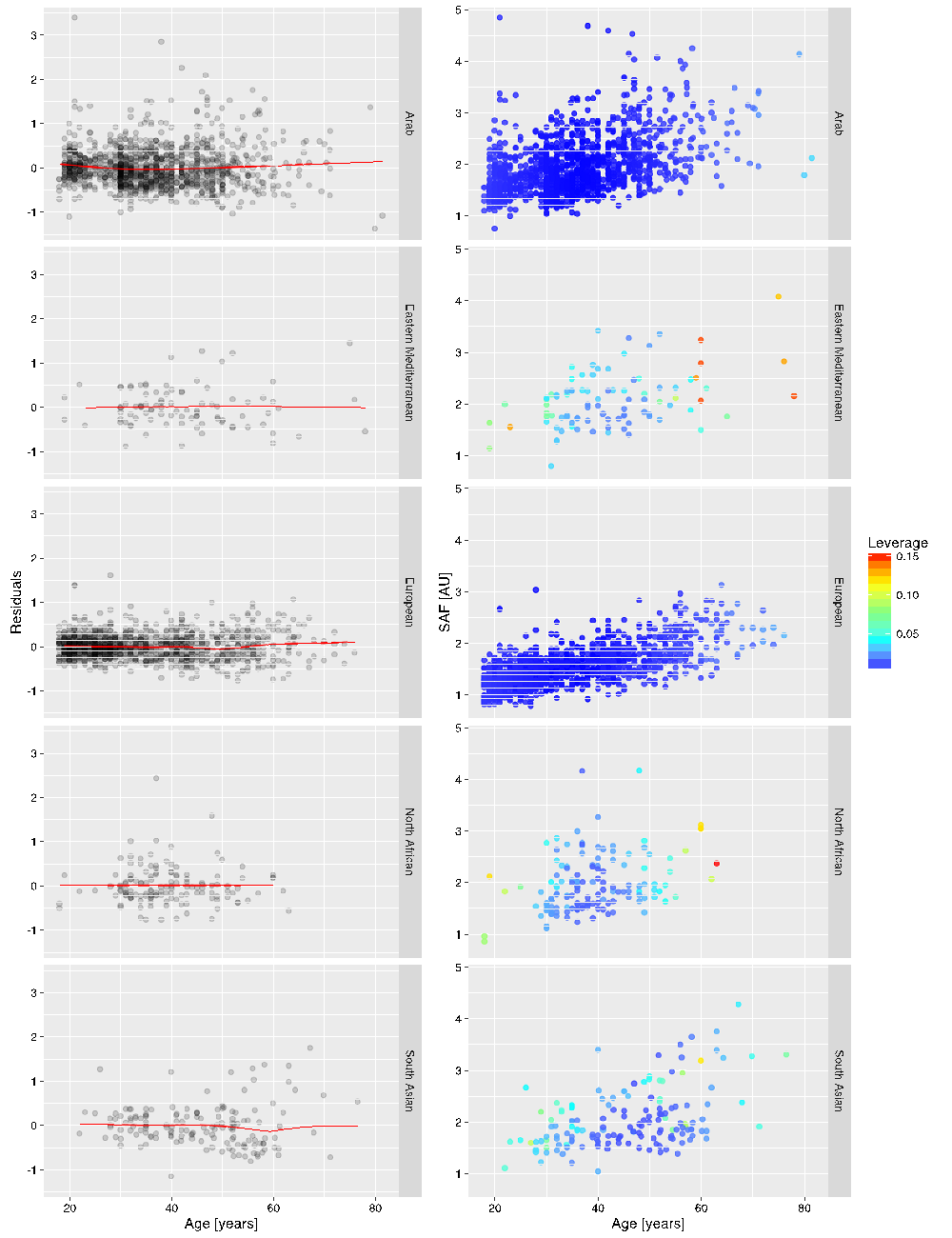

Supplement: S1 Fig — Residual plots (left): all residuals are centred around zero and show a fairly random pattern. The red line is a smoothed approximation of the running mean across 20 observations. Leverage plots (right): Data points with high leverage are coloured towards red in the colour spectrum. (TIF) [file pone.0185175.s001.tif]
