# Supplementary material for: Ethnicity and skin autofluorescence-based risk-engines for cardiovascular disease and diabetes mellitus
Source: PLoS One. 2017 Sep 20;12(9):e0185175. doi: 10.1371/journal.pone.0185175 (PMC5607192; doi:10.1371/journal.pone.0185175)
Supplement: S1 Table — (DOCX) [file pone.0185175.s002.docx]

**S1 Table. Cohort overview.**

| **Description** | **n** | **Reference** |
| --- | --- | --- |
| Cardiovascular disease cohort | 124 | Not published before |
| Diabetes and nutrition cohort | 111 | Not published before |
| Reference population (mainly Arabs) | 1,445 | Ahmad *et al.* [13] |
| Slovak reference population | 1,172 | Klenovics *et al.* [14] |
| Arab and South Asian population | 163 | Mook-Kanamori *et al.* [15] |
