# Supplementary material for: Ethnicity and skin autofluorescence-based risk-engines for cardiovascular disease and diabetes mellitus
Source: PLoS One. 2017 Sep 20;12(9):e0185175. doi: 10.1371/journal.pone.0185175 (PMC5607192; doi:10.1371/journal.pone.0185175)
Supplement: S2 Table — (DOCX) [file pone.0185175.s003.docx]

**S2 Table. Overall cohort baseline characteristics.**

|  | **Healthy**  (n=2,780) | **Diseased**  (n=235) | **P value** |
| --- | --- | --- | --- |
| **SAF** [AU] | 1.70 (0.75-4.84) | 2.8 (1.20-6.92) | 5.7 x 10^-77^ |
| **SR** [AU] | 0.12 (0.04-0.50) | 0.08 (0.06-0.22) | 6.0 x 10^-14^ |
| **Age** [years] | 35 (18-81) | 46 (18-90) | 6.2 x 10^-17^ |
| **Female Gender** | 1,444 (52%) | 108 (46%) | 0.07 |
| **Ethnicity** |  |  |  |
| European | 1,177 (42 %) | - | <<0.001 |
| Arab | 1,181 (42%) | 235 (100%) |  |
| South Asian | 152 (6%) | - |  |
| Eastern Mediterranean | 92 (3%) | - |  |
| North African | 137 (5%) | - |  |
| Southeast Asian | 29 (1%) | - |  |
| Central-East African | 12 (<1%) | - |  |
