# Supplementary material for: Ethnicity and skin autofluorescence-based risk-engines for cardiovascular disease and diabetes mellitus
Source: PLoS One. 2017 Sep 20;12(9):e0185175. doi: 10.1371/journal.pone.0185175 (PMC5607192; doi:10.1371/journal.pone.0185175)
Supplement: S3 Table — (DOCX) [file pone.0185175.s004.docx]

**S3 Table. Healthy cohort overview segmented by country of birth.**

|  |  |  |  |  |  |
| --- | --- | --- | --- | --- | --- |
| **Ethnicity** | **Country of Birth** | **n (%f)** | **Age (yrs)** | **SAF (AU)** | **SR (AU)** |
| **Arab** (n=1,181) | Arab* | 85 (60) | 53.5 (27.31-81.4) | 2.62 (1.42-4.53) | 0.12 (0.06-0.38) |
|  | Kuwait | 8 (50) | 38.6 (33-47) | 2.14 (1.37-3.28) | 0.13 (0.08-0.26) |
|  | Saudi Arabia | 978 (45) | 34.5 (18-80) | 1.94 (0.75-4.84) | 0.09 (0.06-0.25) |
|  | United Arab Emirates | 2 (50) | 31 (29-33) | 2.53 (2.24-2.81) | 0.2 (0.18-0.23) |
|  | Yemen | 108 (39) | 38.9 (21-66) | 1.95 (1.18-3.46) | 0.09 (0.06-0.27) |
| **Central-East African** (n=12) | Eritrea | 2 (100) | 45 (40-50) | 2.77 (2.54-2.99) | 0.07 (0.06-0.08) |
|  | Somalia | 1 (0) | 42 | 1.81 | 0.06 |
|  | Sudan | 9 (22) | 44.4 (37-58) | 1.8 (1.23-2.54) | 0.07 (0.06-0.09) |
| **Eastern Mediterranean** (n=92) | Jordan | 23 (17) | 40.6 (19-65) | 1.94 (0.79-2.75) | 0.1 (0.06-0.15) |
|  | Lebanon | 7 (43) | 41.9 (30-60) | 1.94 (1.44-2.67) | 0.12 (0.07-0.22) |
|  | Palestine | 19 (42) | 50.6 (31-78) | 2.39 (1.49-3.35) | 0.11 (0.06-0.2) |
|  | Syria | 42 (40) | 39.8 (19-76) | 2 (1.13-4.07) | 0.14 (0.07-0.25) |
|  | Turkey | 1 (100) | 35 | 2.4 | 0.29 |
| **European** (n=1,177) | Netherlands | 1 (0) | 32 | 1.62 | 0.09 |
|  | Romania | 1 (100) | 32 | 2.41 | 0.28 |
|  | Slovakia | 1,172 (64) | 34.3 (18-76) | 1.52 (0.79-3.12) | 0.16 (0.04-0.5) |
|  | Spain | 1 (0) | 21 | 1.43 | 0.14 |
|  | UK | 2 (50) | 30 (28-32) | 2.38 (1.74-3.03) | 0.09 (0.09-0.09) |
| **North African** (n=137) | Algeria | 3 (67) | 40.3 (32-46) | 1.9 (1.83-1.95) | 0.16 (0.11-0.24) |
|  | Egypt | 123 (31) | 38.9 (18-63) | 1.95 (0.86-4.16) | 0.1 (0.06-0.29) |
|  | Libya | 1 (100) | 37 | 2.57 | 0.12 |
|  | Morocco | 7 (100) | 42.4 (34-60) | 2.23 (1.75-3.05) | 0.12 (0.1-0.14) |
|  | Tunisia | 3 (0) | 46.7 (36-53) | 1.8 (1.48-1.97) | 0.09 (0.08-0.1) |

| **South Asian** (n=152) | Afghanistan | 2 (100) | 40.5 (36-45) | 2.23 (1.86-2.6) | 0.11 (0.09-0.13) |
| --- | --- | --- | --- | --- | --- |
|  | Bangladesh | 3 (0) | 34 (25-48) | 1.55 (1.22-1.79) | 0.06 (0.06-0.06) |
|  | Burma | 1 (0) | 52 | 1.8 | 0.07 |
|  | India | 35 (23) | 40.6 (22-68) | 1.95 (1.11-3.76) | 0.08 (0.06-0.14) |
|  | Pakistan | 46 (43) | 41.4 (26-61) | 2.07 (1.05-3.5) | 0.09 (0.06-0.15) |
|  | South Asian* | 65 (17) | 52.2 (31.74-76.57) | 2.11 (1.39-4.27) | 0.08 (0.06-0.14) |
| **Southeast Asian** (n=29) | Indonesia | 6 (50) | 45.2 (30-73) | 2.61 (1.7-4.36) | 0.08 (0.06-0.11) |
|  | Malaysia | 1 (100) | 53 | 2.47 | 0.08 |
|  | Philippines | 22 (91) | 45.5 (26-65.4) | 2.41 (1.61-3.81) | 0.11 (0.08-0.16) |

*not further specified.
