# Supplementary material for: Ethnicity and skin autofluorescence-based risk-engines for cardiovascular disease and diabetes mellitus
Source: PLoS One. 2017 Sep 20;12(9):e0185175. doi: 10.1371/journal.pone.0185175 (PMC5607192; doi:10.1371/journal.pone.0185175)
Supplement: S1 Text — (DOCX) [file pone.0185175.s005.docx]

**Inclusion of ethnicity as a predictor variable**

In addition to the linear models obtained for each ethnicity, a single linear model was constructed by including the variable ethnicity and a gender:ethnicity interaction term.

The obtained model’s intercept amounted to 1.4, with an age and male gender coefficient of 0.022 and 0.414 (all p values <2*10^-16^), respectively, which describes the average SAF intensity for individuals with Arab ethnicity (as this ethnicity was represented by the majority of observations). The European population showed less high SAF values (ethnicity coefficient of -0.535, p value <2*10^-16^) and a much lower gender effect (male gender:ethnicity interaction coefficient = 0.344, p value <2*10^-16^). The coefficient for the South Asian ethnicity amounted to -0.277 (p value=0.046), showing that South Asian individuals on average have lower SAF baseline values than individuals of Arab and Eastern Mediterranean ethnicity and higher SAF baseline values than Europeans. The more pronounced gender effect in North African individuals was indicated by a male gender:ethnicity interaction coefficient of -0.194 (p value=0.007). The Eastern Mediterranean population did not significantly differ from the Arab ethnicity, with an ethnicity coefficient of 0.036 (p value=0.820) and a gender:ethnicity interaction coefficient of 0.008 (p value=0.92).

The linear model obtained by including ethnicity and gender: ethnicity interaction term describes the same trends as the linear models created for each ethnicity. For reasons of clarity and comparability, we decided to focus on the description of linear models created for each ethnicity.
